# Typical Exertional Angina With No Angiographic Coronary Artery Disease

**DOI:** 10.7759/cureus.61255

**Published:** 2024-05-28

**Authors:** Yaroslav Zuyev, Tyson Hillock, Rezaul Islam

**Affiliations:** 1 Cardiology, Edward Via College of Osteopathic Medicine, Monroe, USA; 2 Cardiology, St. Francis Hospital, Monroe, USA

**Keywords:** exertion chest pain, junctional bradycardia, atypical chest pain, exertional chest pain, non-cardiac chest pain

## Abstract

Cardiac syndrome X (CSX) is a cardiac condition that is a diagnosis of exclusion. Patients usually present with terrible chest pains suggestive of myocardial infarction, but angiogram imaging shows no occlusion in the coronary vessels that would be suggestive of coronary artery disease. CSX is more commonly seen in women, but this case report demonstrates a different clinical presentation of CSX in a young, otherwise healthy male patient. The 38-year-old male patient presented to the emergency room with chest discomfort radiating to the left arm and to the left jaw. The chest pain started after the patient went for a jog, with the pain lasting for a couple of hours. The electrocardiogram (ECG) was abnormal, showing nonspecific ST changes and unremarkable troponin levels. The patient underwent a coronary angiogram, which was unremarkable. Three years later, the patient presented once more with chest heaviness that occurred again after going for a run. The patient's troponins were unremarkable, and an ECG test showed a new onset of AV block. Due to the ongoing chest pain, the patient received another coronary angiogram. This showed that the coronary vessels had no indications of occlusion. The patient was discharged and scheduled to follow up with their cardiologist for an extensive discussion about medications for their condition. This case report should bring awareness of the classical presentation of this disease in an uncommon population group and a way to identify this syndrome once exclusions have been made on previous hospitalizations.

## Introduction

Chest discomfort can have many causes, ranging from non-life-threatening to life-threatening. When patients present with chest discomfort that radiates to the left arm or jaw, it is important to rule out ST-elevation myocardial infarction (STEMI), and guidelines suggest getting an electrocardiogram (ECG) right away [1]. Coronary artery disease is one of the more serious cardiac diseases. Coronary angiograms have been the gold standard for the diagnosis of coronary artery disease and should be utilized in assessing whether the chest pain has a cardiac origin [1]. When angiogram results show no stenosis, and in the absence of other explanations for these chest pains like STEMI, angina, or reflux, the diagnosis of cardiac syndrome X (CSX) is given as a diagnosis of exclusion [2]. It is estimated that roughly 70% of CSX patients are women [3]. A study found that >60% of those women were postmenopausal [4]. Given the lack of guidelines for treatment for CSX, treatment approaches focus on treating the symptoms and comorbidities. Certain diseases that cause endothelial damage, such as diabetes, hypertension, and hypercholesterolemia, can lead to the development of this rare condition, so it is important to monitor and treat associated conditions [5]. Patients with a CSX diagnosis should follow up with a cardiologist, who should educate the patient on the syndrome and treat the comorbidities.

## Case presentation

**Figure 1:**
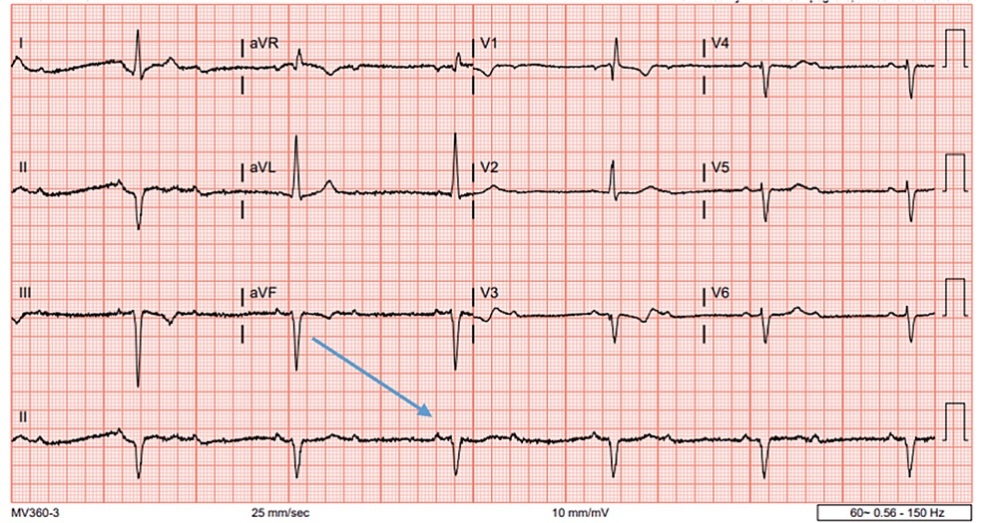
ECG: arrow showing 2:1 atrioventricular block

**Figure 2:**
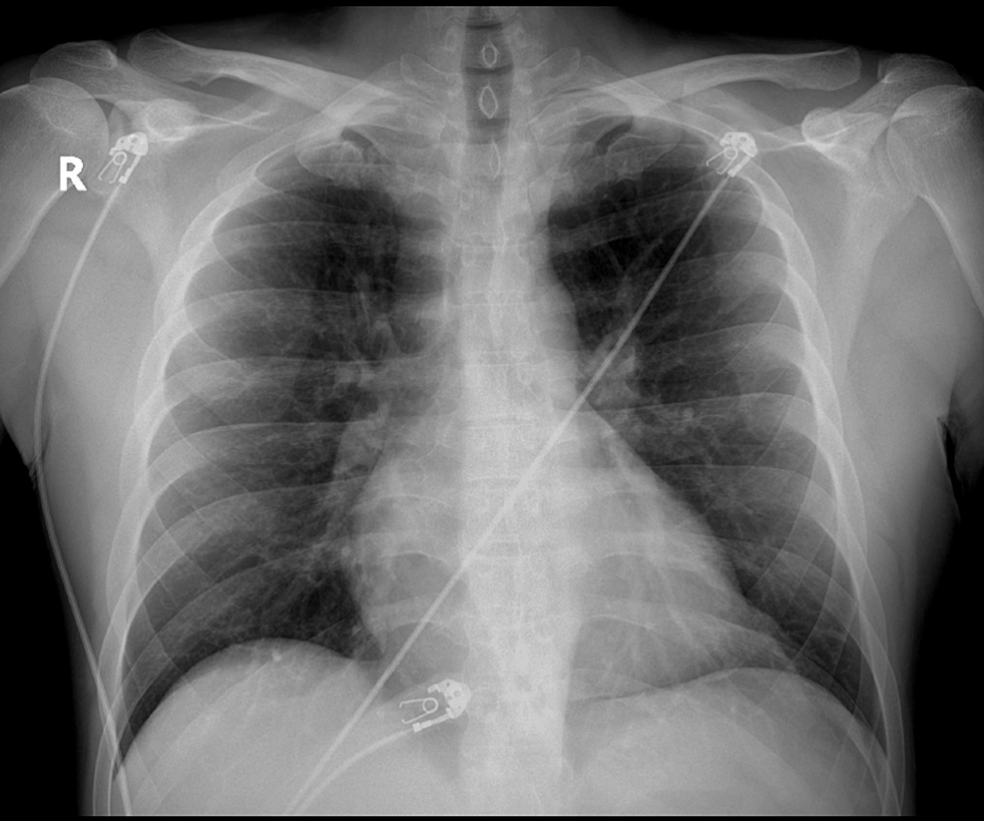
Normal chest X-ray

**Figure 3:**
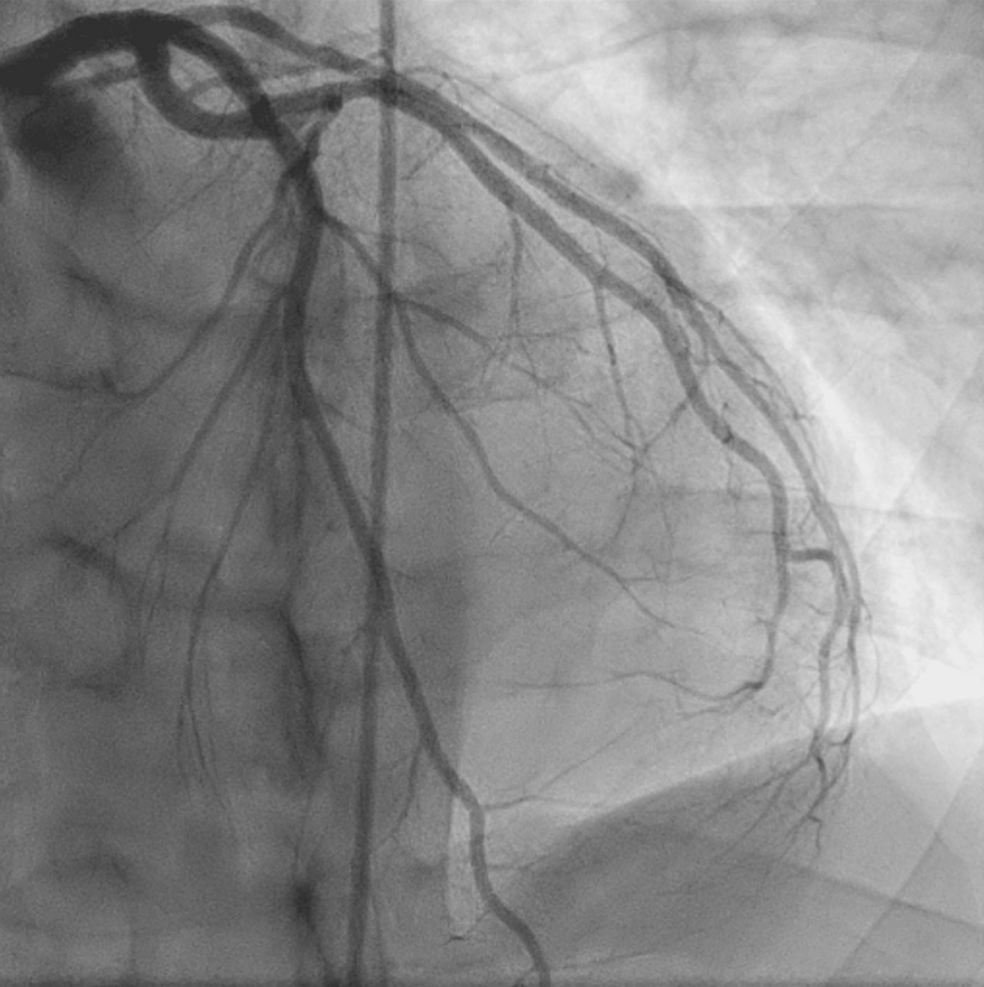
Normal coronary angiogram, showing no signs of obstructions in the left anterior descending artery and left circumflex artery

**Figure 4:**
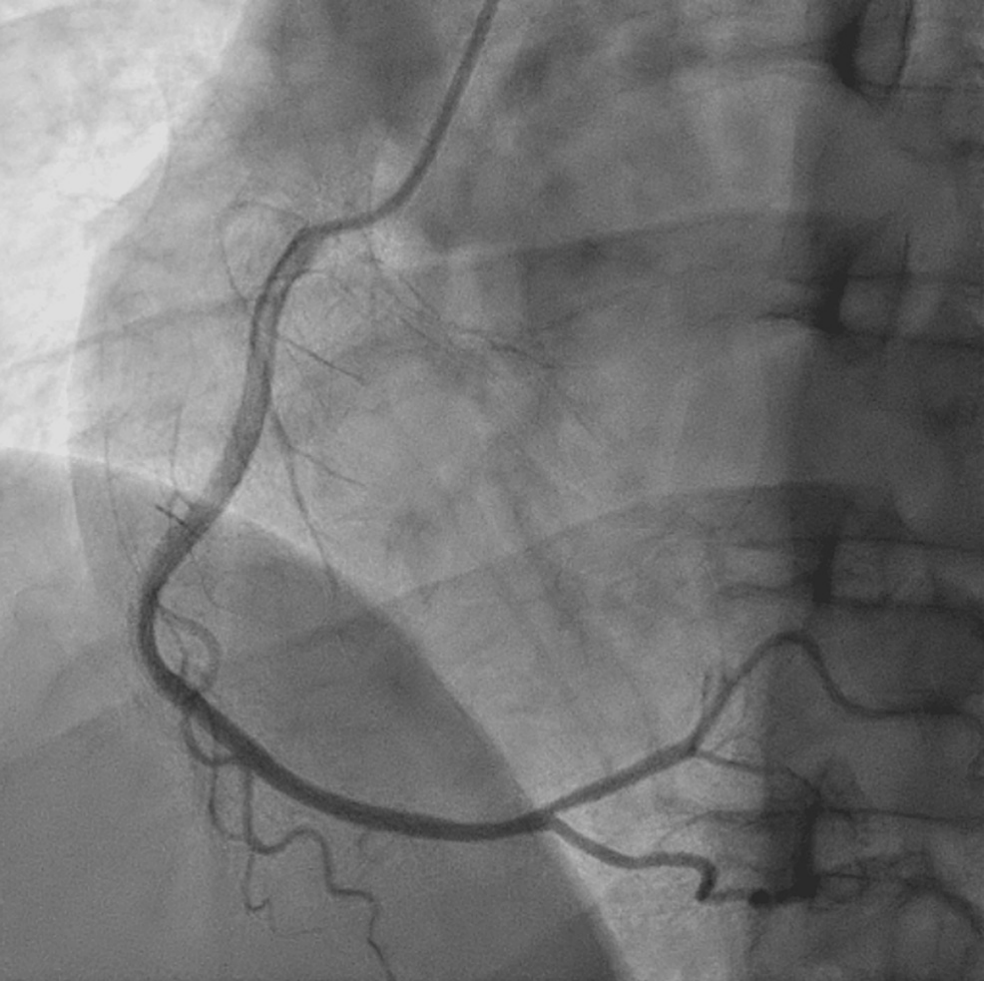
Normal coronary angiogram, showing no signs of obstructions in the right coronary artery

A 38-year-old male presented to the emergency department (ED) with the primary concern of severe chest pressure that radiated to his left arm and jaw; the pressure started after he went for a run and has progressively worsened until he stopped exercising. The patient had a blood pressure of 173/110 and a heart rate of 50 beats per minute. The physical exam at the time was unremarkable. The coronary angiogram did not indicate stenoses or coronary artery disease. The patient was discharged, advised to take a daily aspirin of 81 mg, and told to follow up with a cardiologist. Three years later, the 41-year-old presented to the ED again with chest pressure. After being on a treadmill for 10 minutes, he started having chest pain that did not resolve within a week. The blood pressure on presentation to the ED was 163/79, with bradycardia of 36 beats per minute. The heart rate proceeded to fall to 32 beats per minute, with a heart block on the ECG. Lab results were unremarkable for troponins but showed hypothyroid and a hemoglobin A1C of 6.7% (Table 1). An ECG was performed on the patient and showed a second degree 2:1 AV heart block (Figure 1). The chest X-ray showed no pneumothorax, edema, or effusions and a normal heart size (Figure 2). An X-ray and computed tomography scan (CT) of the chest and head were done to rule out conditions that can cause symptoms, such as pulmonary embolism. We decided, based on the symptoms of chest pain radiating to the left arm and the possibility that the stress testing would result in a false negative, to perform a heart catheterization [6]. The coronary vessels were free of any occlusion, and the image was overall unremarkable (Figures 3-4). Because the angiogram was clear, we decided to implement a pacemaker for the patient's heart block.

**Table 1:** Patients lab values for troponin, hemoglobin A1C, cholesterol, and triglycerides

Laboratory findings		
Lab test	Result	Normal
Troponin	0.01 ng/mL	0–0.04 ng/mL
HbA1C	6.7%	Below 5.7%
Total cholesterol	216 mg/dL	<200 mg/dL
Triglycerides	268 mg/dL	Below 150 mg/dL

The patient had a significant family history of uncontrolled hypertension, hypertriglyceridemia, hypercholesteremia, diabetes, and bradycardia on the father’s side; the mother’s side was unknown. The grandfather and father both had a history of hypertension that was resistant to several medications. The grandfather had a pacemaker implanted when he was in his 30s, for unknown reasons. The patient’s family history of these associated metabolic conditions, such as early diabetes and hypertension, may suggest a genetic component influencing earlier endothelial dysfunction in the cell, even though the patient was maintaining a healthy lifestyle. The patient stated that he did not drink alcohol, use drugs, or smoke and consumed a healthy diet with vegetables and lean meat. The patient had some comorbidities that he was unaware of, such as hypertension, hyperlipidemia, and diabetes, even though he was a fit male who exercised daily.

The patient was discharged and told to follow up with his primary care physician and cardiologist to control symptoms of hypertension and comorbidities. Comorbidities such as hypertension and diabetes are commonly found prior to the diagnosis of CSX. The patient was taking metformin, gemfibrozil, losartan, and amlodipine to control diabetes, hyperlipidemia, and hypertension. After the pacemaker was put in place, the patient was instructed to follow up with the cardiologist within two weeks.

## Discussion

Cardiac syndrome X, being a diagnosis of exclusion, is a rare disorder. Patients typically present with severe chest pain suggestive of coronary ischemia, yet they also have a normal coronary angiogram [5]. Major predisposing factors that can cause this condition include disorders that can lead to endothelial dysfunction, such as hypertension, diabetes, and hypercholesteremia [5]. Endothelial damage is a process that occurs when certain disorders, such as diabetes, cause increased oxidative stress on the blood vessels, leading to damage in the vessels [7]. The presence of predisposing factors in a patient with chest pains and a normal coronary angiogram with no occlusions further suggests the diagnosis of CSX. It is important to assess the patient with a proper history and physical examination, as many of the labs, like the ECG, nuclear stress test, and echocardiogram, may be mostly normal in CSX patients [3]. It was shown in a study of long-term care of CSX patients that chest pain remained in 80% of patients, 137 out of 170 patients, but the prognosis was overall positive, with no major ailments in patients with CSX [8]. In a study of the long-term care of CSX patients, those who did pass away did not do so for cardiac reasons. Hospital readmission for chest pain remained at 58% for CSX [9].

The long-term management of CSX includes lifestyle modification and pharmacological management. It would be recommended to discuss smoking cessation, as this may worsen the syndrome by leading to further endothelial dysfunction [10]. This can include pharmaceutical management of hypertension, diabetes, and hyperlipidemia. It is also important to control diabetes in these patients, as diabetes increases the chances of a myocardial infarction [11]. The best approach for CSX is symptom management and the close monitoring of comorbidities.

## Conclusions

Cardiac syndrome X is an underdiagnosed condition since it is a diagnosis of exclusion. It is important for medical professionals to understand the unique presentation of this disease. Patients with this syndrome overall have a good prognosis, but there needs to be continued management of the patient's symptoms and associated conditions.
